# The Role of Transportation in the Enrollment of Elderly African Americans into Exercise and Memory Study: GEMS Study

**DOI:** 10.1007/s40615-022-01367-7

**Published:** 2022-08-05

**Authors:** Lennox Graham, Julius Ngwa, Oyonumo Ntekim, Oludolapo Ogunlana, Steven Johnson, Sheeba Nadarajah, Thomas V. Fungwe, Jillian Turner, Mara Ramirez Ruiz, Javed Khan, Thomas O. Obisesan

**Affiliations:** 1 Department of Health Management, College of Nursing and Allied Health Sciences, Howard University, Washington, DC 20059 USA; 2 Division of Cardiovascular Medicine, College of Medicine, Howard University, Washington, DC 20059 USA; 3 Department of Nutritional Sciences, College of Nursing and Allied Health Sciences, Howard University, Washington, DC 20059 USA; 4 Department of Medicine and Clinical Translational Science Program, Division of Geriatrics, Howard University Hospital, 2041 Georgia Ave NW, Washington, DC 20060 USA; 5 Department of Nursing, College of Nursing and Allied Health Sciences, Howard University, Washington, DC 20059 USA

**Keywords:** Recruitment, Transportation, African Americans, Clinical research, Mini–Mental State Examination, Memory, Exercise

## Abstract

**Background:**

Understanding the factors driving recruitment and enrollment of African Americans (AA)s in clinical translational research will assure that underrepresented populations benefit from scientific progress and new developments in the diagnosis and treatment of Alzheimer’s disease and related disorders. While transportation is pivotal to volunteers’ ability to participate in research, its contribution to enrollment in exercise studies on AD is yet to be elucidated. Thus, this research focuses on identifying factors that influence the recruitment and enrollment of African Americans in biomedical studies and determining whether the availability of transportation motivates participation in time-demanding exercise studies on AD.

**Methods:**

We analyzed recruitment data collected from 567 volunteers ages 55 and older screened through various recruitment sources and considered for enrollment in our exercise and memory study. To determine whether transportation influenced the enrollment of African Americans (AA)s in biomedical studies, multiple logistic regression analysis was performed to identify significant factors that drive enrollment. Furthermore, the association of race and demographic factors on the availability of transportation was assessed.

**Results:**

Demographic factors, age at screening, education, gender, and cognitive scores were not significantly different among those enrolled compared to control (not-enrolled). In the relationship of enrollment to transportation, enrolled participants were more likely to have access to transportation (79.12%) than not-enrolled participants who had less access to transportation (71.6%); however, the association was not statistically significant. However, race differentially influenced the likelihood of enrollment, with elderly AAs being significantly less likely to have transportation (*p* = 0.020) than the Whites but more likely than “others” to have transportation.

**Conclusion:**

Our findings suggest that access to transportation may be a key factor motivating enrollment in an exercise and memory study in a predominantly AA sample. Notably, AAs in our sample were less likely to have transportation than Whites. Other demographic factors and cognitive scores did not significantly influence enrollment in our sample. A larger sample and more detailed assessment of transportation are needed to further discern the role of transportation in clinical trials.

## Background

Clinical research is necessary to generate evidence for the efficacy and safety of new therapies. Some subgroups of patients may respond differently to medical therapies; for example, women may respond differently than men, and members from one racial or ethnic group may respond differently than those from another [13]. Therefore, a diversity of clinical trial participants is needed to help ensure that the trial population is representative of the patients who will use the medicine or medicinal product and ensure that the results are generalizable [8]. For many decades, ethnic minority groups have historically been underrepresented in clinical trials, a shortcoming that persists in modern trials [27]. Furthermore, because of changing demographics, more than 50% of the US population is projected to be other than non-Hispanic white by 2045 [17]. Congruently, the under-representation of underrepresented ethnic groups in clinical research is a continuing promoter of healthcare disparities in the USA [30]. This under-representation occurs in all types of clinical research and or therapeutic areas, including those diseases that predominantly affect underrepresented groups [7]. Regrettably, the lack of substantive progress affirms that the key parties involved in planning and conducting clinical trials (investigators, sponsors, and regulators) have not fully prioritized inclusion [35]. Upon directives in legislation passed by Congress in the early 1990s, the National Institutes of Health (NIH) instituted policies aimed at increasing the representation of underrepresented populations in clinical trials funded by the agency [5].

The inclusion of diverse participants in clinical research may lead to more robust and complete data that broadens the understanding of racial and ethnic differences in treatment responses, which may contribute to reduced disparities in outcomes [32]. Indorewalla et al. [21] outlined the barriers to recruitment for older adult participants from underrepresented minorities, noting that the need to undergo repeated diagnostic and neurocognitive evaluations, health insurance issues, lack of adequate transportation to and from the medical facility, and high costs of transportation burden participants and deter them from enrolling in ongoing clinical trials [21].

Transportation has been shown to play a significant role in the recruitment and enrollment of AA in biomedical studies. Rivers et al. [31] reported that important impediments to successful recruitment of AA to clinical trials included negative attitudes towards clinical trials, low levels of knowledge and awareness regarding clinical trials, and religious beliefs. Importantly structural barriers such as transportation, childcare, and access to healthcare [31] were also noted. The authors acknowledged that AA patients are more likely to have transportation problems getting to their medical appointments than non-Hispanic White patients. Furthermore, the authors added that key strategies that may improve the recruitment and enrollment of AA in clinical trials include resources for transportation to screening appointments, parking, and media advertising [6, 31]. According to Baquet et al. [4], important determinants for participation in clinical trials include lack of transportation, especially for AA females [4]. It is important to note that an exercise study requires multiple visits/weeks over several months. Transportation, therefore, would be critical to volunteers’ ability to participate in clinical trials. Underrepresented minorities may have less transportation due to social-economic means, education, and income [21]. Therefore, we examined factors that influence enrollment of AAs in biomedical studies and determined whether the availability of transportation motivates increased participation. In addition, we examined the association of race and demographic factors on the availability of transportation in motiving participation in these time-demanding exercise studies on memory.

## Methods

The Institutional Review Board (IRB) of Howard University approved the protocols used for this study. As part of the requirement for human subject studies, participants completed a signed consent form before enrolling in the study. In addition, a detailed description of the gene, exercise, and memory study (GEMS) has been published.

### Screening Study Population

We analyzed recruitment data consisting of potential volunteers ages 55 or older engaged at various recruitment sources. Engaged participants included those contacted or prescreened (no signed consent form); enrolled participants included those that were consented and screened; not-enrolled included those that consented but screen-failed. Volunteers completed a Mini–Mental State Exam (MMSE) as part of screening and recruitment for this study. The screening eligibility of the participants consisted of the ability to exercise vigorously without causing harm to self, no chronic medical condition, and met the Petersen MCI criteria [29], which include the following: age and education adjusted score 24–30 inclusive, have objective memory loss, and memory complaints. After completing the informed consent, demographic and general medical history were obtained from the volunteers. Participants who completed the intervention and confirmed to be MCI were randomly assigned to a 6-month program of either aerobic or stretch exercise. These participants underwent 40 min of supervised training three times/week. VO2Max was determined at baseline and repeated after volunteers completed the 6-month exercise program. Most participants used public transportation for the visits, and only a few owned means of transportation.

During the recruitment period, the Division of Geriatrics organized, sponsored, and participated in many health promotion events to educate a predominantly AA community on the warning signs, identification, treatment, and lifestyle changes to reduce the risk of developing AD. These efforts occurred at geriatric and other medical clinics, church events, health fairs, senior housing and assisted living facilities, and senior wellness centers. Additional community engagement activities occurred during Annual NBC News Expo and Annual Congressional Black Caucus Health Event and Christmas gatherings; engagement of Pastoral Leadership Group; through Direct mailing, Newspaper Advertisements; Hospital and Community-Based Flyers; and Hospital Billboard. Study volunteers were not compensated for initial engagements and screenings but received stipends based on completed study visits. The community and leadership groups were first contacted through third parties or an introductory letter, followed by telephone calls.

### Recruitment Strategies

The community outreach program included interactions with several organizations at yearly events and others less systematic but sporadic. During these events, attendees received education on memory disorders, neurodegeneration, and general health concerns common to the geriatric populations. In addition, the principal investigator provided education on risk factors for AD, progress on diagnosis, currently available treatments, and promising preventive strategies. As part of the outreach program, we created sustained alliances. The program includes a Community Alliance for Research Engagement (CARE) group component. This group comprised church leaders in the Washington, DC, metropolitan area who met monthly with the PI and our community outreach team to develop the best strategies for raising awareness in their respective communities. Educational discussions focused on preventing and treating high blood pressure, diabetes, high cholesterol, and memory loss. These alliances occurred mostly in the Howard University geographic region in the District of Columbia and adjacent Prince George’s County, Maryland, that house a predominantly Black population.

Also, the Division of Geriatrics partnered with the Howard University community outreach program and participated in health fairs. Such programs included general health screenings, engagement of seniors and their caregivers, and education on various geriatrics syndromes, including memory loss and AD. We also emphasized the importance of healthy living and emerging preventive strategies for AD. For example, the Washington, DC, Department of Health has over six Senior Wellness Centers where seniors 60 years and older interact and socialize. These centers offer lunch, outings/excursions, computer training, nutrition classes, treadmill exercise, massage, and seminars on health issues affecting their groups and provided education on medication management, health, and other social services issues. The HU community outreach team visited these centers and engaged the attendees through seminars and memory screenings using the MMSE and logical memory to access study eligibility.

## Statistical Analysis

To assess the characteristics of the recruited participants by enrollment status, we used descriptive statistics. All analyses were performed using SAS version 9.3 [22] and NCSS Statistical Software [19]. We assessed variables of interest from the Exercise Study for data distribution and the assumption of normality. From this number, twelve participants were excluded at level 1 screening. Of the remaining 555 participants, 62 participants were excluded due to MMSE scores below 24 and age below 55. The remaining data included 493 participants (enrolled = 92; met general inclusion criteria, signed consent form and undergone additional screening; not-enrolled = 401). The baseline descriptive analyses expressed numerical variables as mean and standard deviation or as the number of participants and proportions when categorical. We evaluated for significant differences using Student’s *t* test for continuous variables and employed a chi-square test to analyze categorical data. We present a boxplot distribution of the age at screening and enrollment status as well as gender categories, comparing the enrolled participants vs. those not-enrolled (those that were not enrolled). In addition, we performed logistic regression analyses to inform the role of transportation and identify demographic and cognitive factors that affected enrollment. All *p* values were based on a two-tailed test with significance set at *p* < 0.05 and confidence intervals computed at a 95% confidence level.

## Results

### Enrollment of Engaged Volunteers

A total of 567 senior residents in the DELMARVA area were engaged as potential volunteers in the exercise study. Of the 567 engaged volunteers, 555 had level 1 screenings, while thirteen had incomplete screening data. Inclusion criteria required participants to have an MMSE score ≥ 24 and aged 55 and above. This resulted in a total of 493 with screening data and 62 excluded from the analysis (inclusion criteria, MMSE scores ≥ 24 and age ≥ 55). Overall, 92 (18.66%) signed the study informed consent, while 401 (81.34%) were not-enrolled because they did not meet the enrollment criteria or were lost to follow-up.

### Characteristics of Engaged Volunteers

**Fig. 1:**
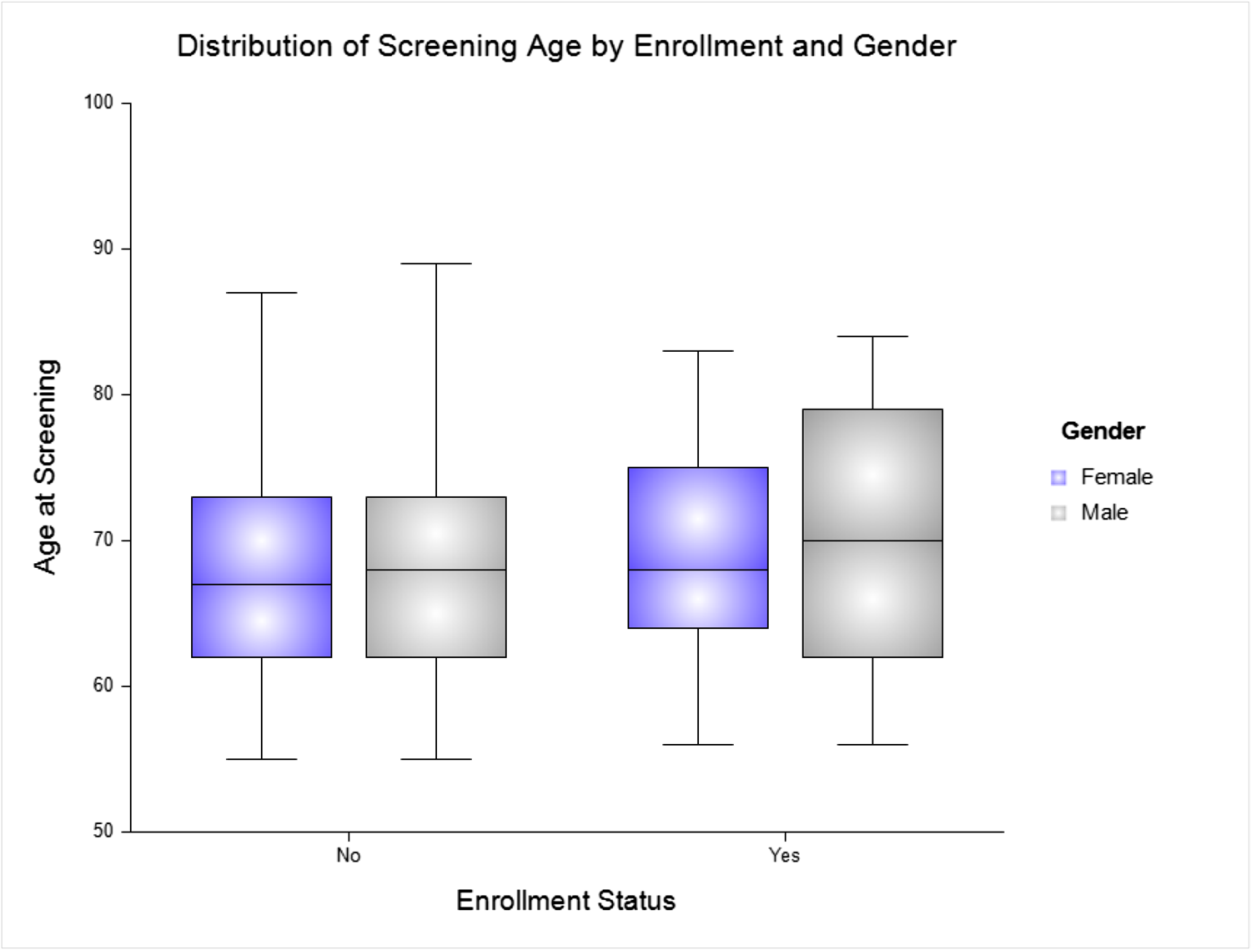
Boxplot of age at screening by enrollment status (enrolled vs. not-enrolled) by gender (male and female)

Among the 493 engaged and considered for the study, 81.34% were AAs or Blacks, 9.33% White, and 6.29% considered other race (Table 1). The mean age of the overall sample at screening was 68.27 (7.57), with enrolled participants being significantly older (mean age 69.67±7.46; *p* = 0.048) compared to the not-enrolled (mean age = 67.95±7.57). Figure 1 shows the distribution of the screening age by enrollment and gender. Among the enrolled and not-enrolled groups, women constituted a higher percent of the sample (74.24%). Further, the mean MMSE score was significantly higher (*p* = 0.009) among the enrolled (28.43±1.33) than the not-enrolled (28.00±1.72) participants. The samples were relatively similar in years of educational attainment and logical memory scores (LM1 and LM2).

**Table 1:** Characteristics of participants (*N* = 493)

	Engaged (*N* = 493)	Enrolled (*N* = 92)	Not-enrolled (*N* = 401)	*p* value
Age screen	68.27 (7.57)	69.67 (7.46)	67.95 (7.57)	0.048
Gender (% female)	366 (74.24%)	65 (70.65%)	301 (75.06%)	0.362
Transportation	279 (73.42%)	72 (79.12%)	207 (71.63%)	0.158
Education (yrs.)	12.11 (5.50)	12.21 (4.81)	12.08 (5.66)	0.829
Race				
Blacks	401 (81.34%)	90 (97.83%)	311 (77.56%)	< 0.001
Whites	46 (9.33%)	0 (0.00%)	46 (11.47%)	
Others	31 (6.29%)	2 (2.17%)	29 (7.23%)	
MMSE	28.08 (1.66)	28.43 (1.33)	28.00 (1.72)	0.009
Logical memory 1	10.09 (3.85)	10.18 (3.45)	10.06 (4.01)	0.777
Logical memory 2	7.82 (3.60)	7.73 (3.03)	7.86 (3.82)	0.754

Continuous data expressed as mean (SD); categorical data as frequencies (%); categorical variables compared using Fisher exact test and continuous variables using the Student’s *t* test

Given the trend in transportation, the characteristics of participants by enrollment and transportation status were examined. In Table 2, we present the characteristics of the participants by enrollment and transportation. Among enrolled participants, those with transportation were slightly older (mean age 70.58±7.05) than those with no transportation (mean age 66.53±8.36), but the difference did not reach statistical significance (*p*=0.064). The enrolled participants were relatively similar to the not-enrolled in gender, years of educational attainment, MMSE, and logical memory scores (LM1 and LM2). However, as anticipated and among the not enrolled participants, those with transportation were significantly more educated (mean years of education=13.07±5.53) than those without transportation (mean years of education 10.74±5.36) (*p*=0.001). Notably, not-enrolled participants with transportation performed better on the MMSE cognitive test and cognitive scores than those without transportation.

**Table 2:** Characteristics of participants by enrollment and transportation (*N* = 493)

	Enrolled (*N* = 92)			Not enrolled (*N* = 401)		
	No transport (*N* = 19)	Transport (*N* = 72)	*p* value	No transport (*N* = 82)	Transport (*N* = 207)	*p* value
Age screen	66.53 (8.36)	70.58 (7.05)	0.064	68.05 (7.69)	68.51 (7.29)	0.640
Gender (% female)	14 (73.68%)	50 (69.40%)	0.786	62 (75.61%)	154 (74.40%)	0.882
Education	11.47 (5.28)	12.39 (4.73)	0.499	10.74 (5.36)	13.07 (5.53)	0.001
Race						
Blacks	17 (89.47%)	72 (100.00%)	0.042	67 (81.71%)	166 (80.19%)	0.087
Whites	0 (0.00%)	0 (0.00%)		6 (7.32%)	29 (14.01%)	
Others	2 (10.53%)	0 (0.00%)		9 (10.98%)	11 (5.31%)	
MMSE	28.37 (1.46)	28.46 (1.31)	0.809	27.77 (1.87)	28.19 (1.59)	0.072
Logical memory 1	11.00 (4.32)	9.97 (3.21)	0.343	9.53 (3.89)	10.68 (4.00)	0.071
Logical memory 2	8.21 (3.47)	7.61 (2.94)	0.497	7.48 (3.54)	8.28 (3.83)	0.178

Continuous data expressed as mean (SD); categorical data as frequencies (%); categorical variables compared using Fisher exact test and continuous variables using the Student’s *t* test

**Fig. 2:**
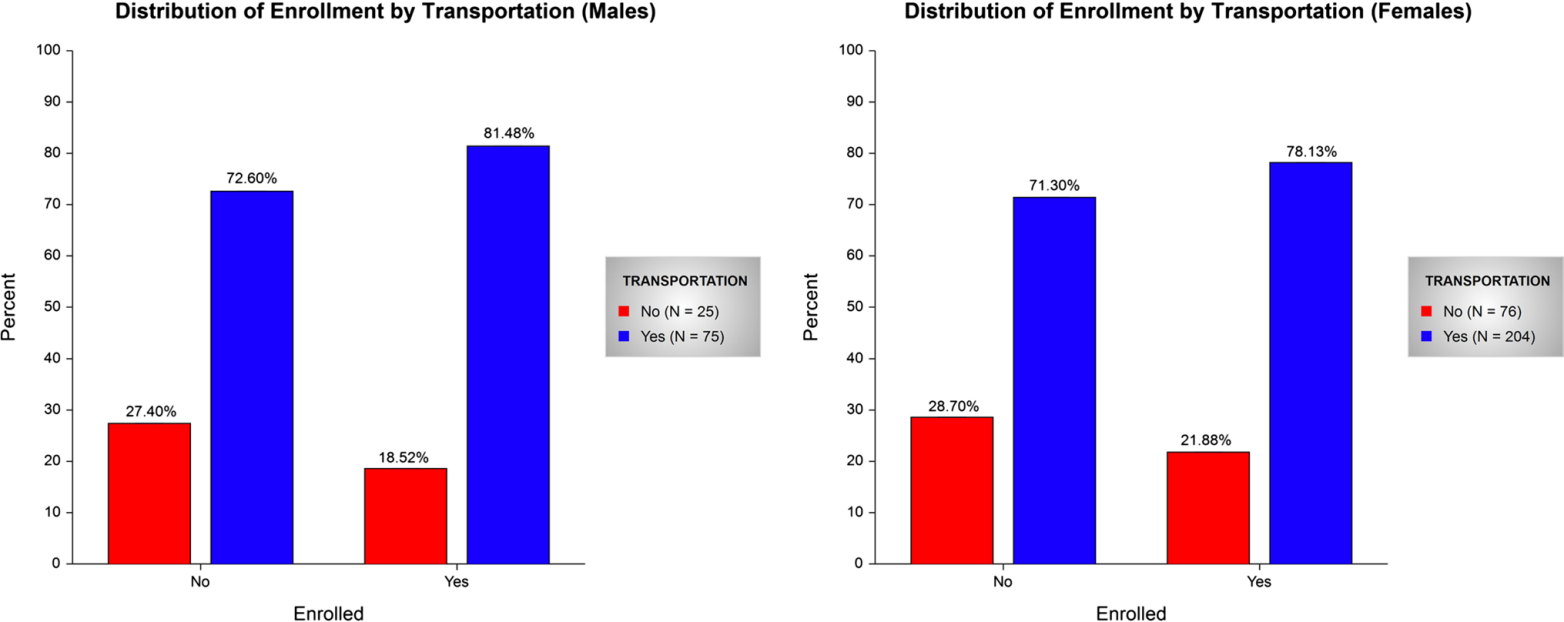
Frequency distribution of enrollment (enrolled vs. not enrolled) by transportation (yes or no) stratified by gender (male and female)

Interestingly, a higher percent of the enrolled participants (79.12%) had access to transportation than the not-enrolled (71.63%). Figure 2 shows the enrolled vs. not-enrolled frequency distribution by transportation and gender (male vs. female). Among female participants, a total of 204 (72.86%) volunteers had transportation while 76 (27.14%) did not, and among male participants, 75 (75.00%) had transportation while 25 (25.00%) did not. Among enrolled female participants, enrolled participants were more likely to have transportation (78.13%) than not-enrolled (71.30%). Conversely, a higher percent (81.48%) of enrolled men had transportation than not-enrolled (72.60%).

### Factors Associated with Enrollment

We performed logistic regression analysis to assess the relationships of enrollment status with demographic factors and cognitive scores (Table 3). In the model, age (at screening), education, gender, and cognitive scores (LM1 and LM2) were not significantly different among those enrolled compared to those not-enrolled. However, differences in access to transportation were not statistically significant (*p* = 0.088) between groups. Participants who had access to transportation were more likely to be enrolled.

**Table 3:** Association of demographic factors and enrollment

Characteristics	Estimate	Std. error	*z* value	*p* value
Age at screening	0.013	0.018	0.693	0.488
Transportation	0.540	0.316	1.708	0.088
Gender (male vs. female)	0.019	0.290	0.064	0.949
Education	−0.020	0.027	−0.738	0.461
Logical memory 1	0.025	0.060	0.425	0.671
Logical memory 2	−0.043	0.064	−0.677	0.498

Logistic regression: association of enrollment with demographic factors and cognitive scores

### Factors Associated with Transportation

We performed a logistic regression analysis to evaluate potential demographic and race factors that may influence transportation in our sample (Table 4). In the unadjusted model, AAs were less likely to have means of transportation compared to the white volunteers (*p* = 0.035). Additionally, participants categorized as “others” (Asian, Hispanic, Islander Latino, and Native Americans) combined due to small sample were less likely to have transportation compared to the AAs (*p* = 0.007). The associations remain consistent after adjusting for age at screening, gender, and education, with AAs still less likely to have transportation than Whites (*p* = 0.020) but more likely to have transportation than others (*p* = 0.003). Participants with higher levels of education were more likely to have transportation as well (*p* = 0.0006).

**Table 4:** Association of demographic factors, race, and transportation

Unadjusted analysis				
Characteristics	Estimate	Std. error	Wald statistics	*p* value
Race				
Whites vs. Blacks	0.703	0.334	4.439	0.035
Others vs. Blacks	−0.873	0.324	7.251	0.007
Adjusted analysis				
Characteristics	Estimate	Std. error	Wald statistics	*p* value
Race				
Whites vs. Blacks	0.817	0.352	5.393	0.020
Others vs. Blacks	−1.047	0.356	8.662	0.003
Age at screening	0.020	0.017	1.510	0.219
Gender (female vs. male)	−0.071	0.140	0.257	0.612
Education	0.142	0.042	11.871	0.0006

Logistic regression: association of transportation and race adjusting for demographic factors

Others in race include Asian, Hispanic, Islander, Latino, and Native Americans

## Discussion

This study examined factors influencing enrollment into a biomedical study in a predominantly AA sample, primarily focusing on transportation. Among the demographic factors, age at screening, education, gender, and cognitive scores (logical memory 1 and 2) were not significantly different among those enrolled compared to those not-enrolled. Overall, transportation was not statistically significantly associated with enrolled status though more enrolled participants had access to transportation than not-enrolled participants. However, an important finding was that enrolled AAs were less likely to have transportation than “Whites” but more likely than “others” category. Similarly, education appeared to be a proxy marker for transportation -- Those with higher levels of education have more transportation.

Our study included a predominantly AA sample with more women than men volunteers. Graham et al. [14] noted that particularly, AA women are motivated to participate in health-related research because of altruism, monetary, and other compensations [14]. Our current study focused on elderly AA population, average age ~70 years. Notably, enrolled volunteers were relatively more educated and performed better on the cognitive assessment tasks (MMSE scores) than the not-enrolled group. Interestingly, those having transportation were more educated (higher years of education) than those without transportation in the not-enrolled group. Potentially, this may have been motivated by a greater sense of altruism and willingness to participate in research among the older (older old) versus younger (young–old) volunteers. Further, not-enrolled participants with transportation (have transportation) performed better on the MMSE than those with no transportation (have no transportation), suggesting that driving and navigating may have cognitive benefits. An interesting and potentially cognitive benefit of navigating ability is supported by Maguire et al. [26], who showed that structural MRIs of the brains of humans with extensive navigation experience (licensed London taxi drivers) had significantly larger posterior hippocampi than control subjects [26].

While the enrollment of elderly AAs into studies on Alzheimer’s disease and related disorders remains relatively low, the role of transportation in the under-enrollment of this population has not been fully elucidated. An important observation from this study is that AAs in our sample were less likely to have transportation than Whites. This indicates that transportation may be an important mediator of lower participation of AAs compared to Whites in clinical translational research. Few studies have examined the role of transportation as a conduit to participation in research. For example, Fox [10], in a survey to assess the need for transportation infrastructure, contacted 843 clinical trial sites in the USA to understand how transportation access impacted participant recruitment and enrollment and the type of transportation participants found most helpful. Of the 49 sites that responded, 95% reported that transportation infrastructure would improve recruitment efforts, and 63% felt it would ensure all studies recruited on time. Eighty-four percent of respondents reported that a taxi or ride-sharing service partnership would be the preferred transportation infrastructure. Also, Frank et al. [11] indicated that individuals who live farther from the clinical trial site seem more likely to accept rides, suggesting that transportation infrastructure may help clinical trial sites engage with harder-to-reach populations and broaden their recruitment networks [11]. These observations are congruent with our findings that transportation may be a likely catalyst to study enrollment. Unfortunately, the importance of transportation is yet to be fully appreciated by study teams. For example, Sertkaya et al. [34] pointed out that coordinators spend minimal time and effort coordinating rides, though patient satisfaction surveys suggest that the service would greatly benefit study participants [34]. Thus, education of staff together with the provision of transportation services may yet enhance study enrollment and reduce attrition, especially in under-resourced AA and other underrepresented groups.

While acknowledging known barriers, we must continuously strive to identify hidden ones and develop new strategies to overcome them. The barriers and influencers of enrollment in biochemical trials among low income, disadvantaged AA groups have been reported by other groups [4, 12, 16, 18, 20, 23, 33, 36]. Given the acknowledged barriers, efforts to dismantle these impediments to scientific progress must be deliberate and continuous. Notably, the under-representation of racial and underrepresented groups would limit the generalizability of research findings. In addition, under-enrollment of racial and ethnic underrepresented groups in clinical trials may also contribute to preventable disparities in treatment outcomes and survival [5]. Amorrortu et al. [3] suggested the need for recruitment strategies that facilitate referrals from physicians outside of the specialty clinics who may see a higher proportion of underrepresented groups of patients [3]. This approach will allow a broader cross-section of these groups to be exposed to available clinical trials. In a survey of 70,000 research volunteers, Alexander-Bridges and Doan [2] reported that underrepresented volunteers were just as likely as majority volunteers to participate in clinical research when approached by their own physicians [2]. Furthermore, while it is believed that underrepresented volunteers fail to participate in trials because of distrust for research and researchers, others have suggested that an additional limiting factor is that underrepresented groups are not routinely asked to participate [9] in clinical studies. Nonetheless, our findings from this study suggest that lack of transportation is yet another puzzle in the barriers to participation in clinical research.

Other factors that are not characterized as transportation may yet serve as proxies for transportation, influence volunteers satisfaction and delay enrollment and timely completion of clinical studies. Estimates of the number of trials that fail to meet scientific objectives because of insufficient accrual rates range from 22 to 50% [27]. Low accrual rates jeopardize the ability of researchers to assess the safety and effectiveness of new approaches to medical care, waste resources, delay follow-up studies, and translation of research into evidence-based practice [25]. Despite the National Institutes of Health requirement that members of underrepresented populations be included in clinical research [15], under-enrollment from lack of transportation is even more significant for economically disadvantaged vulnerable populations such as African Americans. Other factors such as the attitude of research staff have been noted to influence volunteers satisfaction. Also, Adler et al. [1] reported that satisfaction and positive attitudes of research staff and specific trials were important determinants for enrollment, completion, and participation in clinical trials. Additionally, given the importance of cultural competence to the successful design and implementation of a given study, Otado et al. [28] suggested that research teams should consist of multiethnic staff, involve the community, demonstrate trust, and deliver concise education to prospective study volunteers [28]. In addition to lack of transportation to clinic locations for study visits, Kalbaugh et. al. [24] noted that others were unable to accommodate schedule visits. The most significant gap was seen for AA and Hispanics with less than a high school education and people earning less than $25,000 annually. This result is consistent with our findings that AAs are less likely to have the transportation needed to enable their participation in clinical studies. Our finding from the current study suggests that targeting older AA with access to transportation may increase recruitment given that the convenience of transportation would promote compliance. Alternatively, study budgets that include support and provision for transportation or embedding clinical trial centers in the community may help ameliorate the barriers posed by lack of transportation. Some of the limitations of this study included the fact that we did not examine the role of cultural sensitivity, compensation, missing demographic data, and other factors that may additionally influence enrollment and retention. We hope to investigate these factors in future studies.

## Conclusion

Our findings suggest transportation may be a key factor mediating the recruitment and enrollment of elderly AAs in an exercise study on AD. Particularly, elderly AAs and other underrepresented minority groups who may live farther from the clinical trial site may be lacking transportation and perhaps more inclined to accept rides. Therefore, a transportation infrastructure may help clinical trial sites engage harder-to-reach populations. The availability and convenience of transportation may broaden recruitment networks, mitigate study recruitment and enrollment, and promote compliance. A larger sample is needed to further discern the role of transportation in clinical trials on AD.

## Abbreviations

AA: African Americans
AD: Alzheimer’s disease
GEMS: Gene, exercise, and memory study
LM: Logical memory
MMSE: Mini–Mental State Examination
CARE: Community Alliance for Research Engagement
